# Catastrophic health expenditure of households with hypertension: a comparative study in China

**DOI:** 10.3389/fpubh.2023.1176170

**Published:** 2023-06-08

**Authors:** Xiaohui Zhai, Zhongliang Zhou, Guanping Liu, Jiao Lu, Yaxin Zhao, Dan Cao, Dantong Zhao, Sha Lai, Xiaojing Fan

**Affiliations:** ^1^School of Public Health, Health Science Center, Xi’an Jiaotong University, Xi’an, Shaanxi, China; ^2^School of Public Policy and Administration, Xi’an Jiaotong University, Xi’an, Shaanxi, China

**Keywords:** hypertension, catastrophic health expenditure, multimorbidity, propensity score matching, China

## Abstract

**Objectives:**

The aim of this study was to understand the impact of multimorbidity on catastrophic health expenditures for people with hypertension.

**Methods:**

Data were obtained from the China Health and Retirement Longitudinal Study (CHARLS) in 2018, 8,342 adults were included in our analysis. Propensity score matching method was used to compare the risk of catastrophic health expenditures between the hypertension patients (treatment group) and those without any chronic disease (control group) in middle-aged and older adults. Patients with hypertension were also divided into two groups: only hypertension and multimorbidity.

**Results:**

Hypertension increased the likelihood of CHE by 11.3% in older adults. Further analysis showed that hypertension alone does not increase the risk of CHE, and the risk of CHE in hypertension patients with multimorbidity was 12.9% higher than those without chronic disease.

**Conclusion:**

Our study highlights the importance of healthy management of patients with only hypertension and preventing them from developing multimorbidity.

## Background

Hypertension has a high prevalence worldwide. Approximately 31% of adults worldwide have hypertension and another 25–50% have pre-hypertension (1). According to the latest report, the number of people aged 30–79 with hypertension has doubled globally over the past 30 years, from 648 million in 1990 to 1.28 billion in 2019 (2). Hypertension is a common and dangerous chronic disease and a major risk factor for death worldwide, causing 10.8 million deaths in 2019 (3). 82% of people with hypertension live in low-income and middle-income countries. Among them, China is one of the countries with the highest increase in the prevalence of hypertension in the past 30 years (2). With the aging population, approximately 245 million Chinese suffered from hypertension in 2017, and 2.54 million died of high systolic blood pressure, 95.7 percent of whom died from cardiovascular diseases (4).

Hypertension not only poses a serious health risk to individuals in China but also imposes a heavy economic burden on society and the families of patients. In 2013, the direct economic burden of hypertension in China was 210.34 billion yuan (about $33.95 billion), accounting for 6.6% of the total health expenditure (4). Hypertension is incurable and has no obvious symptoms in its early stages. Sometimes the management of hypertension becomes a late treatment of complications or acute exacerbations, which is more expensive (5, 6). This imposes a heavy financial burden on the families of patients and even leads to catastrophic health expenditure (CHE), especially among the poor (7).

Multimorbidity is defined as the presence of two or more chronic diseases in the same person at the same time (8), which has led to increased medical costs and raised the risk of CHE (9–12). Previous studies on multimorbidity patterns have shown that multimorbidity is most common in hypertensive patients (13, 14). Many studies have assessed CHE and health service utilization in hypertensive patients with multiple chronic conditions (15, 16). For example, a study conducted in rural China showed that patients with hypertension who have complications were at a higher risk of CHE (10). But few studies have estimated the risk of CHE for people with only hypertension among the older adults in China.

In addition, the risk of CHE is unequally distributed among people of different economic levels (17). Many studies have examined and compared CHE at different household economic levels. The results of relevant studies are inconsistent and in some cases contradictory. Most studies have shown that higher economic level was associated with a lower risk of CHE (18, 19), while a study in Malawi found that the poorest quintile of households had a lower incidence of CHE and were at lower risk of CHE compared to middle-income and wealthier households (20). Another study indicated that households with the lowest economic status faced similar risks of CHE as those with the highest economic status, and households with the middle economic status had lower risks of CHE (21).

Overall, patients with hypertension may have a higher risk of CHE, and whether the impact of hypertension on CHE varies by economic level is unclear. Therefore, the aim of this study was to investigate the effect of hypertension on CHE in middle-aged and older adults. First, we compared CHE between the full sample of hypertensive patients and the population without chronic disease to identify the impact of having hypertension on CHE in middle-aged and older adults. Taking into account multimorbidity, we divided hypertensive patients into two groups: the hypertension only group and the multimorbidity group. We estimated their risk of CHE compared to the group without chronic disease, respectively. Further, we explored the heterogeneity of this effect in different economic level groups. The findings of this study have important implications for strengthening China’s medical insurance policy and improving the chronic disease management of middle-aged and older adults.

## Methods

### Data source

Data used in this study were obtained from the China Health and Retirement Longitudinal Study (CHARLS). CHARLS is a nationally representative longitudinal survey conducted by Peking University and followed up every 2 years (22). It aims to collect a set of high-quality microdata representing the families and individuals of middle-aged and older adults in China to meet the need for scientific research on older people. A stratified multi-stage and probability proportional to size (PPS) sampling were used in the project. The survey data were obtained by trained investigators using the computer-assisted personal interview system instead of paper questionnaires. In order to ensure sample representability, the CHARLS baseline survey covered 150 districts and counties, 450 villages and urban communities, involving 17,708 people in 10,257 households. The survey in 2018 included 752 districts and counties. It reflects the overall situation of China’s middle-aged and older adults.

**Figure 1:**
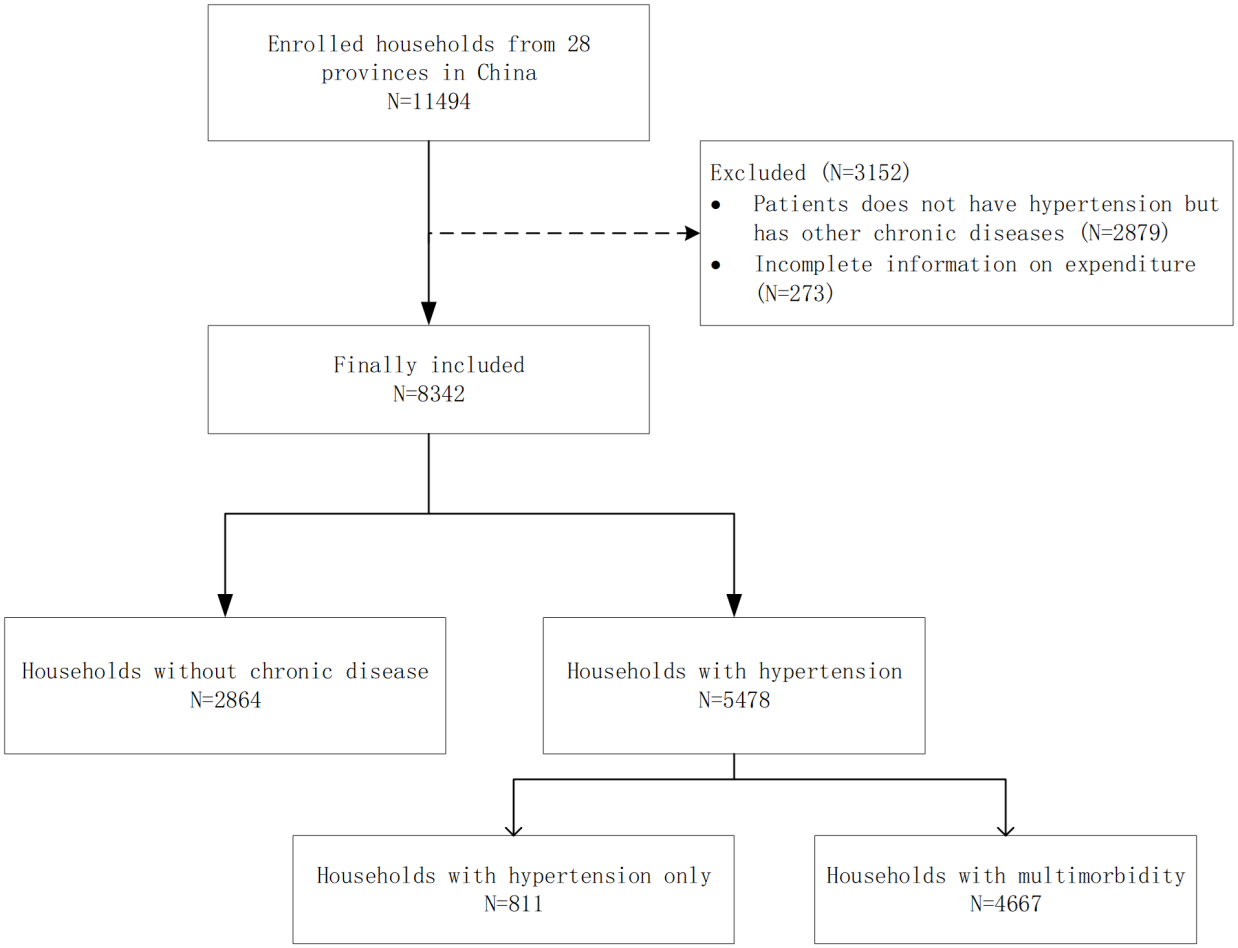
Sampling flow chart.

This study used the follow-up data from 2018. We processed the original data as follows: First, to control for the confounding effects of other diseases, the samples were restricted to those without any chronic diseases and those with hypertension, and patients with hypertension were classified as having only hypertension and multimorbidity (i.e., one or more chronic diseases in addition to hypertension). Second, observations that lacked important spending information were deleted. The number of deleted samples was 3,152. Finally, a total of 8,342 households were included in this study. (Flow chart of sample selection, Figure 1).

### Variables

#### Dependent variable

##### Measurement of CHE

Catastrophic health expenditure is considered to have occurred when the share of health expenditure in a household’s capacity to pay exceeds a certain threshold. However, there is still controversy over the threshold of CHE in academic fields. According to existing research, the point at which health-care costs become catastrophic ranges between 10 and 50% of household expenditures. In this study, when out-of-pocket healthcare expenditure equals to or exceeds 40% of a household’s capacity to pay (CTP), it is considered catastrophic (23). Out-of-pocket payments (OOP) include payments for health-related services during the previous 12 months. It does not include the part already paid by medical insurance. CTP is calculated by subtracting food expenditure from total household expenditure (24). CHE is a dichotomous variable and can be calculated according to the following formula:


(1) $$E_{i}=\{\begin{array}{c} 1,if\;OOP/CTP\geq z\\ 0,if\;OOP/CTP<z \end{array}$$

Where E_i_ represents whether the household has experienced CHE, z is the threshold of CHE, and the z value in this study is 0.4. The incidence and intensity of household CHE can be calculated according to E_i_.


(2) $$Headcount=\frac{1}{N}\overset{N}{\underset{i=1}{\sum }}E_{i}$$

(3) $$Overshoot=\frac{1}{N}\overset{N}{\underset{i=1}{\sum }}E_{i}\left(\left(OOP/CTP\right)-z\right)$$

(4) $$MPO=\frac{1}{M}\overset{N}{\underset{i=1}{\sum }}E_{i}\left(\left(OOP/CTP\right)-z\right)$$

In formula (2) (4), N is the sample size. M is the number of households that incurred CHE. Headcount represents the incidence rate of CHE. Overshot and MPO represent the intensity of CHE. Overshot reflects the severity of CHE for all households in the community, and MPO indicates the intensity of the impact of CHE on households where CHE has occurred.

### Grouping variable

#### Assessment of hypertension and multimorbidity

A question of “Have you been diagnosed with conditions listed below (including hypertension, dyslipidemia, diabetes, cancer, chronic lung diseases, liver disease, heart problems, stroke, kidney disease, digestive diseases, psychiatric problems, memory-related diseases, arthritis and asthma) by a doctor?” was used to measure multimorbidity in this study. Based on the respondents’ answers, we divided them into three groups: without chronic disease, only hypertension and multimorbidity, and those with multimorbidity was defined as the presence of one or more chronic diseases in addition to hypertension.

### Covariates

A range of demographic and socioeconomic variables were included as covariates, such as gender; age group (45 ~ 59, 60 ~ 69, 70 ~ 79 and ≥ 80 years); education (illiteracy, primary and below, junior to senior high and junior college and above); marital status (married, else); employment status (employed, unemployed); household size (≤2, 3 ~ 4 and ≥ 5 people); health insurance [none, urban employee basic medical insurance (UEBMI), urban–rural resident medical insurance (URRBMI) and else]; having disabled members in household (no, yes); inpatient service use (no, yes); outpatient service use (no, yes); residence (rural, urban); region (eastern, central and western); and economic level (Q1 ~ Q5). Economic levels were measured by *per capita* household expenditure rather than income (25). We divided into five economic levels based on the quintile of household consumption expenditure *per capita*.

### Statistical analysis

Descriptive statistics were used to summarize the characteristics of the sample. All categorical variables were described by number and frequency. A chi-square test was used to compare the distribution of the various independent variables between the person that without chronic disease, hypertensive only and hypertensive patients with multimorbidity.

In order to solve the problem of selection bias, we used the propensity score matching (PSM) method to estimate the economic burden of hypertension on the household economy. PSM is a statistical method proposed by Rosenbaum & Rubin (1983) (26) for making causal inferences from non-experimental data. The basic idea is to use a “counterfactual” design to form an approximate random experimental scenario. The mean treatment effect was estimated by calculating the mean difference between the treatment and control groups. In this study, the older adults with hypertension were the treatment group. To improve the accuracy of the estimation, we divided hypertensive patients into two subgroups: hypertensive only and hypertensive patients with multimorbidity. The older adults without any chronic disease were the control group. For the selection of covariates, we adopted a strategy recognized by previous studies, that is, all covariates that may be related to outcome variables are included in the model, regardless of whether they are related to grouped variables (27). The matching result is considered acceptable if the standardization deviation is less than or equal to 20% (26). We used the Logit model to predict the propensity score for hypertension in older adults and matched the propensity score to estimate the average treatment effect on the treated (ATT), and the 95% confidence interval of the ATT value was estimated by bootstrapping. Finally, different matching algorithms, including radius matching, nearest neighbor matching and kernel matching, were used to test the robustness of the results.


(5) $$ATT=E\left(Y_{1i}\mathrm{|,}H_{i}=1\mathrm{|,}p\left(x\right)\right)-E\left(Y_{0i}\mathrm{|,}H_{i}=0\mathrm{|,}p\left(x\right)\right)$$

Where P(x) is the propensity score estimated by logistics regression, which indicates the conditional probability of a person being assigned to a particular study condition (treatment or control) under the conditions given by other control variables. Y_1i_ and Y_0i_ represent the CHE of the older adults in the treatment group and the control group, respectively. H is the indicator variable of whether the older adults have hypertension. H_i_ = 1 for the hypertension group and H_i_ = 0 for the group without chronic disease.

We performed a stratified analysis to identify differences in CHE among patients of different economic levels. The statistical analyses were performed using Stata 17. And *p* < 0.05 was considered statistically significant.

## Results

### Demographic characteristics

Table 1 presents the socio-demographic characteristics of the respondents by health condition. Among the 8,342 samples, 2,864 (34.33%) did not have any chronic diseases, 811 (9.72%) only suffered from hypertension, and 4,667 (55.95%) suffered from multimorbidity. Most of the respondents were married (79.33%), employed (76.68%), lived in rural areas (59.09%) and had no disabled family members (62.74%). 17.18% had received inpatient care in the past year, and 15.60% had used outpatient services during the last month. The proportions of gender, age group, education, employment status, household size, having disabled members, inpatient service use, outpatient service use, region and economic level differ significantly across different groups (*p* < 0.05). The distribution of people’s residence and health insurance in the three groups did not differ significantly (*p* > 0.05).

**Table 1:** Characteristics of respondents by health conditions [*n* (%)].

socio-demographic characteristics	Total	Households with hypertension (*n* = 5,748)		Without chronic diseases (*n* = 2,864)	*p*
		Only hypertension (*n* = 811)	Multimorbidity (*n* = 4,667)		
Gender					**<0.001**
Male	3,998(47.93)	419 (51.66)	2,128(45.60)	1,451(50.66)	
Female	4,344(52.07)	392 (48.34)	2,539(54.40)	1,413(49.34)	
Age group (years)					**<0.001**
45 ~ 59	3,434(41.17)	332 (40.94)	1,344(28.80)	1758(61.38)	
60 ~ 69	2,729(32.71)	249(30.70)	1739(37.26)	741 (25.87)	
70 ~ 79	1,568(18.80)	163(20.10)	1,148(24.60)	257 (8.97)	
80~	611(7.32)	67(8.26)	436(9.34)	108 (3.77)	
Education					**<0.001**
Illiteracy	1952(23.40)	188(23.18)	1,247 (26.72)	517 (18.05)	
Primary and below	3,546(42.51)	355(43.77)	2004 (42.94)	1,187 (41.45)	
Junior to Senior High	2,658(31.86)	253(31.20)	1,324 (28.37)	1,081 (37.74)	
Junior college or above	186(2.23)	15(1.85)	92 (1.97)	79 (2.76)	
Marital status					**<0.001**
Married	6,618(79.33)	648 (79.90)	3,515 (75.32)	2,455 (85.72)	
Else 1	1724(20.67)	163 (20.10)	1,152 (24.68)	409 (14.28)	
Work status					**<0.001**
Employed	5,100(61.14)	529 (65.23)	2,375 (50.89)	2,196 (76.68)	
Unemployed	3,242(38.86)	282 (34.77)	2,292 (49.11)	668 (23.32)	
Household size					**<0.001**
≤2	4,084(48.96)	390 (48.09)	2,401 (51.45)	1,293 (45.15)	
3 ~ 4	2,430(29.13)	242 (29.84)	1,247 (26.72)	941 (32.86)	
≥5	1828(21.91)	179 (22.07)	1,019 (21.83)	630 (22.00)	
Health insurance					0.198
None	265(3.18)	24 (2.96)	142 (3.04)	99 (3.46)	
UEBMI	1,116(13.38)	101 (12.45)	666 (14.27)	349 (12.19)	
URRBMI	6,391(76.61)	634 (78.18)	3,538 (75.81)	2,219 (77.48)	
Else 2	570(6.83)	52 (6.41)	321 (6.88)	197 (6.88)	
Residence					0.075
Rural	4,929(59.09)	498 (61.41)	2,709 (58.05)	1722 (60.13)	
Urban	3,413(40.91)	313 (38.59)	1958 (41.95)	1,142 (39.87)	
Disabled members					**<0.001**
No	5,234(62.74)	576 (71.02)	2,417 (51.79)	2,241 (78.25)	
Yes	3,108(37.26)	235 (28.98)	2,250 (48.21)	623 (21.75)	
Inpatient service use					**<0.001**
No	6,907(82.82)	743 (91.62)	3,448 (73.91)	2,716 (94.83)	
Yes	1,433(17.18)	68 (8.38)	1,217 (26.09)	148 (5.17)	
Outpatient service use					**<0.001**
No	7,039(84.40)	733 (90.38)	3,667 (78.61)	2,639 (92.14)	
Yes	1,301(15.60)	78 (9.62)	998 (21.39)	225 (7.86)	
Region					**<0.001**
Eastern	3,162(37.90)	342 (42.17)	1,599 (34.26)	1,221 (42.63)	
Central	3,135(37.58)	288 (35.51)	1859 (39.83)	988 (34.50)	
Western	2045(24.51)	181 (22.32)	1,209 (25.91)	655 (22.87)	
Economic level					**<0.001**
Q1 (the lowest)	1,669(20.01)	187 (23.06)	1,014 (21.73)	468 (16.34)	
Q2	1,673(20.06)	160 (19.73)	967 (20.72)	546 (19.06)	
Q3	1,664(19.95)	147 (18.13)	944 (20.23)	573 (20.01)	
Q4	1,668(20.00)	158 (19.48)	869 (18.62)	641 (22.38)	
Q5 (the highest)	1,668(20.00)	159 (19.61)	873 (18.71)	636 (22.21)	

Else 1 includes unmarried, divorced and widowed. URRBMI includes Urban Resident Basic Medical Insurance (URBMI) medical insurance and New Cooperative Medical Scheme (NCMS). Else 2 includes government medical insurance and private medical insurance.

### Incidence and intensity of CHE

**Figure 2:**
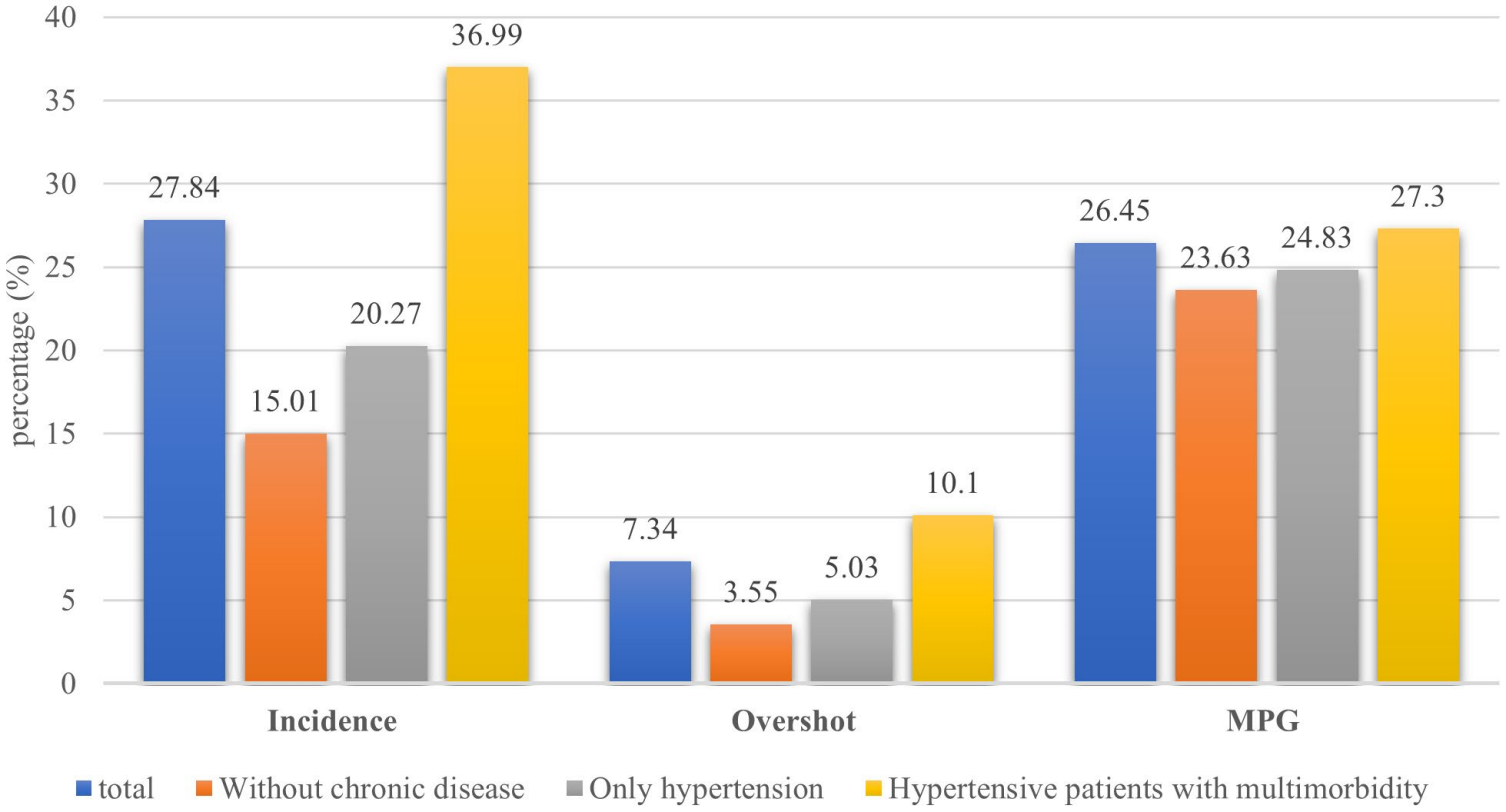
Incidence and intensity of CHE.

Figure 2 presents the incidence and intensity of CHE for different groups. The overall incidence of CHE among the samples was 27.84%, the overall overshoot and MPG of the samples were 7.34 and 26.45%, respectively. The incidence of CHE is lowest among people without chronic disease (15.01%) and highest among hypertension patients with multimorbidity (36.99%). Similarly, the overshoot and MPG were lowest among people without chronic disease, at 3.55 and 23.63%, respectively. These rates were highest in those hypertensive patients with multimorbidity, at 10.1 and 27.3%, respectively.

### Balancing tests

The balancing tests of this study are shown in Table 2. Before matching, there were statistically significant differences in all covariates except health insurance and residence between the hypertension group (full sample) and the group without chronic disease. After matching, there was no significant difference in marital status, working status, household size, health insurance, residence, disabled members, and region between the two groups. The number of covariates with statistically different decreased, and the maximum absolute standardized difference was less than 10%, indicating that matching effectively reduced the difference in covariates between the two groups. Similarly, covariables in subgroups were well balanced after matching. (See Supplementary Table S1).

**Table 2:** Differences in covariates before and after matching.

variables	Mean			STD (%)	*t*-value
		Patients with hypertension (full sample)	Without chronic diseases		
Gender	U	0.464	0.507	−8.5	−3.69***
	M	0.469	0.503	−6.8	−3.51***
Age	U	2.115	1.548	64.6	27.27***
	M	2.072	2.149	−8.8	−4.00***
Education	U	2.066	2.256	−24.3	−10.48***
	M	2.080	2.030	6.3	3.20***
Marital status	U	1.239	1.141	25.4	10.65***
	M	1.231	1.244	−3.4	−1.60
Work status	U	0.530	0.768	−51.4	−21.68***
	M	0.545	0.542	0.5	0.23
Household size	U	1.709	1.771	−7.8	−3.37***
	M	1.712	1.725	−1.7	−0.87
Health insurance	U	2.868	2.879	−2.0	−0.86
	M	2.867	2.908	−7.1	0.000
Residence	U	0.415	0.399	3.1	1.36
	M	0.413	0.404	1.8	0.93
Disabled members	U	0.453	0.217	51.6	21.73***
	M	0.438	0.436	0.4	0.18
Inpatient service	U	0.235	0.052	54.1	21.55***
	M	0.211	0.195	4.7	2.05**
Outpatient service	U	0.197	0.079	34.8	14.26***
	M	0.183	0.161	6.6	3.05***
Region	U	1.900	1.802	12.5	5.43***
	M	1.890	1.889	0.2	0.09
Economic level	U	2.922	3.156	−16.7	−7.20***
	M	2.928	2.842	6.1	3.12***

U denotes before matching, M denotes after matching. STD denotes standardized difference. The tests in the table were based on radius matching and the radius was set to 0.01. *p < 0.1, **p < 0.05, ***p < 0.01.

### The impact of hypertension on CHE

As shown in Table 3, different matching algorithms were used to analyze the effect of hypertension on CHE across different groups. After adjusting for covariates between older adults with hypertension (full sample) and those without chronic disease, the risk of CHE was significantly different between the two groups. The results showed that the risk of CHE was 11.3% (radius matching) higher in older adults with hypertension than in those without chronic disease.

**Table 3:** Estimated effects of hypertension on CHE across different samples.

outcome	Matching algorithm		Full sample of hypertension patients (1)		Only hypertension (2)		Multimorbidity (3)	
			ATT	95% CI	ATT	95% CI	ATT	95% CI
CHE	Before matching		0.195*** (0.010)	–	0.053*** (0.015)	–	0.220*** (0.010)	–
	After matching	Radius	0.113*** (0.014)	[0.085, 0.141]	0.017 (0.016)	[−0.014, 0.049]	0.129*** (0.016)	[0.098, 0.160]
		NN	0.095*** (0.019)	[0.057, 0.132]	0.026 (0.024)	[−0.021, 0.073]	0.125*** (0.020)	[0.087, 0.163]
		Kernel	0.116*** (0.014)	[0.088, 0.144]	0.021 (0.016)	[−0.010, 0.051]	0.131*** (0.015)	[0.101, 0.160]

Radius represents radius matching with the radius of 0.01. NN represents nearest neighbor matching (one-to-one matching). Kernel represents kernel matching with the bandwidth of 0.06. 95% CI were estimated using bootstrap (500 reps). Standard errors in parentheses. *p < 0.1, **p < 0.05, ***p < 0.01.

Further subgroup analysis results showed that there was no significant difference in CHE between those with only hypertension and those without chronic disease, except for the unadjusted analysis. All the algorithms came to the same conclusions, indicating that patients with only hypertension did not increase the risk of CHE. In addition, hypertension patients with multimorbidity significantly increased the risk of developing CHE, with a 12.9% increased risk compared to people without chronic disease.

The ATT values of different matching algorithms were similar, indicating that the results were robust.

### Heterogeneous analysis of the risk of CHE in different economic groups

Table 4 shows the risk of CHE for middle-aged and older adults with hypertension in different economic groups. In the full sample of hypertensive patients (column 1), hypertension was associated with an increased risk of CHE in the low and medium economic levels, and the risk of CHE was 7.4–20.1% higher among hypertensive patients than among those without chronic disease, but this association was not observed in the highest economic level.

**Table 4:** Estimated average treatment effects on the treated (ATT) of hypertension on CHE across different economic level.

Economic level	Matching algorithm	Full sample (1)		Only hypertension (2)		Multimorbidity (3)	
		ATT	95% CI	ATT	95% CI	ATT	95% CI
Q1	Radius	0.159*** (0.036)	[0.088, 0.229]	−0.029 (0.048)	[−0.123, 0.064]	0.207*** (0.038)	[0.131, 0.282]
	NN	0.162*** (0.043)	[0.079, 0.246]	−0.018 (0.065)	[−0.145, 0.109]	0.211*** (0.048)	[0.116, 0.306]
	Kernel	0.166*** (0.034)	[0.100, 0.232]	−0.021 (0.042)	[−0.104, 0.062]	0.193*** (0.036)	[0.123, 0.263]
Q2	Radius	0.162*** (0.032)	[0.100, 0.225]	0.033 (0.044)	[−0.052, 0.119]	0.198*** (0.034)	[0.131, 0.266]
	NN	0.201*** (0.037)	[0.128, 0.273]	0.001 (0.059)	[−0.115, 0.116]	0.211*** (0.041)	[0.130, 0.293]
	Kernel	0.165*** (0.030)	[0.107, 0.223]	0.029 (0.040)	[−0.049, 0.107]	0.190*** (0.032)	[0.128, 0.253]
Q3	Radius	0.077** (0.033)	[0.012, 0.142]	−0.036 (0.043)	[−0.121, 0.049]	0.085*** (0.033)	[0.020, 0.150]
	NN	0.074* (0.040)	[−0.003, 0.152]	−0.014 (0.060)	[−0.132, 0.103]	0.056 (0.042)	[−0.025, 0.138]
	Kernel	0.075** (0.031)	[0.014, 0.035]	−0.032 (0.040)	[−0.110, 0.045]	0.088*** (0.031)	[0.027, 0.148]
Q4	Radius	0.110*** (0.029)	[0.053, 0.166]	0.080** (0.039)	[0.002, 0.157]	0.117*** (0.033)	[0.053, 0.182]
	NN	0.133*** (0.036)	[0.063, 0.203]	0.114** (0.052)	[0.012, 0.216]	0.145*** (0.039)	[0.068, 0.223]
	Kernel	0.123*** (0.026)	[0.072, 0.173]	0.088** (0.038)	[0.013, 0.162]	0.128*** (0.030)	[0.069, 0.186]
Q5	Radius	0.035 (0.026)	[−0.017, 0.086]	0.019 (0.029)	[−0.037, 0.075]	0.014 (0.029)	[−0.042, 0.071]
	NN	0.041 (0.033)	[−0.023, 0.104]	0.006 (0.039)	[−0.070, 0.082]	−0.007 (0.036)	[−0.078, 0.063]
	Kernel	0.041 (0.026)	[−0.011, 0.092]	0.036 (0.027)	[−0.019, 0.086]	0.041 (0.028)	[−0.015, 0.097]

ATT, the average treatment effect on the treated. ATT were presented after performing PSM and bootstrap. Standard errors in parentheses. *p < 0.1, **p < 0.05, ***p < 0.01.

Except for Q4, there was no significant difference in CHE at all economic levels between patients with only hypertension and those without chronic diseases (column 2). The risk of CHE was lower in Q4 than in multimorbidity.

The results also show that at low economic levels, middle-aged and older people with hypertension and other chronic diseases are more likely to incur CHE. For example, in the lower economic levels (Q1 and Q2), the risk of CHE was 19–21% higher for hypertension patients with multimorbidity than for those without chronic disease. In the middle economic level (Q3 and Q4), hypertension patients with multimorbidity had an 8.5–14.5% higher risk of CHE than those without chronic disease. However, at the highest economic level, we did not find a higher risk of CHE among hypertension patients with multimorbidity (column 3).

## Discussion

In this study, we used PSM to compare the risk of CHE among patients with hypertension and those without chronic disease. We contributed to the explanation of this issue by not only focusing on the full-sample effects of hypertensive patients, but also assessing these effects through subgroup analysis. We found that, in general, people with hypertension had an 11.3% higher risk of developing CHE than people without chronic disease. However, there were unequal effects across different subgroups. Concretely, hypertension patients with multimorbidity were at a higher risk of CHE. Only one chronic condition, hypertension, did not increase the risk of CHE. The risk of CHE was also different across different economic levels. The above results were still robust after using different matching algorithms.

Our study found that the incidence of CHE was 27.84% in the sample population, 20.27% in those with only hypertension, and 36.99% in those with multimorbidity. It is slightly higher than the reported 26.46% among community-dwelling older adults in the study based on CHARLS 2013 (28). A study in rural Shaanxi province found that the incidence of CHE in only hypertensive patients and those with multimorbidity was 23.48 and 34.01%, respectively, which was also lower than the incidence of CHE in this study (7). The possible explanation is that the OOP in other studies does not include indirect health expenditure, whereas our study includes both direct and indirect health expenditure (7, 10). In addition, the samples included in this study were middle-aged and older adults aged 45 and above. Previous studies have shown that older age is a risk factor for CHE (29–31), thus the incidence of CHE was higher in this study.

As expected, multimorbidity in patients with hypertension was associated with a higher risk of CHE, which is consistent with other studies (32, 33). In our study, the risk of CHE was 12.9–13.1% higher in patients with multimorbidity than in patients without chronic diseases. Previous studies have shown that multimorbidity is associated with increased rates of consultations, prescriptions, and hospitalizations (34, 35), as well as higher health care expenditures (36). For instance, in the Swiss study by Caroline B et al. (35), the average total cost of a multimorbid patient is 5.5 times greater than a non-multimorbid patient. Each additional chronic disease adds $2,383 to the total annual cost. Multimorbidity increase health expenditures by increasing utilization of health services. Multimorbidity patients also have a higher risk of CHE. Other studies on the multimorbidity of specific diseases have found similar conclusions (11, 12), suggesting that more attention should be paid to the management of multimorbidity to reduce the economic burden of diseases.

Surprisingly, the results of this study showed no significant difference in the risk of CHE between patients with only hypertension and people without any chronic disease. This may be due to the fact that patients with only hypertension have relatively low medical expenses compared to other common chronic diseases (37). A study conducted in Colombia also found that OOP related to hypertension accounted for 1.6 percent of total household expenditures, and it consists mainly of direct non-medical costs, including dietary requirements for non-pharmacological treatment and transportation costs for medical treatment. OOP for hypertension treatment is modest, and CHE is uncommon (38). Other studies have also shown that hypertension patients without complications have a lower risk of CHE (10, 39). However, many studies have estimated the annual cost of hypertension using nationally representative data. At the national level, the attributable costs associated with hypertension are considerable and increasing year by year (40, 41).

Another possible reason for the relatively low risk of CHE for patients with only hypertension is that health management of hypertension reduces the financial burden on patients. Since 2009, China has been implementing the National Basic Public Health Service Project, which is the most basic public health service project provided free of charge to all residents. Health management for hypertensive patients is one of the important elements of this project. In order to control the development of hypertension, health management services for hypertensive patients are provided free of charge by urban and rural primary health care institutions, including screening of high-risk groups, face-to-face follow-up visits and testing four times a year, and classified interventions based on patients’ conditions (42). The standardized management of hypertension patients in the past 10 years has reduced the growth rate of cardiovascular disease mortality and alleviated the economic burden of patients (43).

Furthermore, this study discovered that hypertension patients with multimorbidity in lower economic levels were more likely to develop CHE than those without any chronic disease, whereas this association is not statistically significant in the highest economic level. Previous studies in Kenya (44), Zambia (45), Bangladesh (19), and China (46, 47) have reported similar results. The possible explanation is that the same amount of health expenditure is a higher proportion of CTP for people at lower economic levels. However, this proportion is smaller for those with the highest economic level. A study in Malawi yielded the opposite result: the higher the economic level, the higher the incidence of CHE. This result may be explained by the fact that poor families do not use health services due to the high cost of health care (20).

Our study has several limitations that should be noted. First, the health care spending information in the survey is self-reported, so it is hard to avoid the problem of recall bias. Second, in the process of matching, we only control for observable variables, and if there are unobservable covariates, there will be a “hidden bias.” Third, the chronic diseases included in this study were mainly determined by self-reported information. 14 chronic diseases were asked about, but some common chronic diseases (such as chronic lymphocytic thyroiditis and osteoporosis) were still ignored, so the incidence of multimorbidity may be underestimated. Finally, the severity of the disease is unclear due to data limitations. Future studies may consider rating the severity of disease by incorporating information from physicians’ clinical diagnoses, which is an important confounding factor. Besides, only those over 45 years of age were included in this study, the economic burden of hypertension in younger age groups needs further investigation. Finally, follow-up on the economic burden of disease in patients with hypertension is needed.

## Conclusion

In summary, there was no significant difference in the risk of CHE between patients with only hypertension and those without chronic disease. Hypertension patients with multimorbidity are at higher risk of CHE than those without chronic disease and patients with only hypertension. Furthermore, people in the low economic level group were more prone to CHE. These findings may have implications for us to reduce the economic burden of disease in patients with hypertension. On the one hand, we should continue to promote standardized management of hypertension in the community, and prevention should be given priority to prevent the deterioration of hypertension or cause other diseases. On the other hand, policymakers should develop targeted medical assistance programs for the older adults, especially those with low economic levels, to reduce the economic burden caused by multiple chronic diseases.

## Data availability statement

Publicly available datasets were analyzed in this study. This data can be found at: https://charls.pku.edu.cn/en/.

## Ethics statement

The medical ethics committee approved the CHARLS study, informed consent was signed by all interviewees. Ethics approval for the data collection in CHARLS was obtained from the Biomedical Ethics Review Committee of Peking University (IRB00001052–11015).

We further confirm that all methods were carried out in accordance with relevant guidelines and regulations.

## Author contributions

XZ designed the study, performed statistical analysis, and wrote the manuscript. ZZ, GL, and JL contributed to study design, data analysis, and revision of the manuscript. ZZ, YZ, DZ, DC, SL, and XF made valuable revisions to the original manuscript. All authors contributed to the article and approved the submitted version.

## Funding

This study was funded by leading talents project in philosophy and social sciences of the National Social Science Foundation of China (2022LJRC02).

## Conflict of interest

The authors declare that the research was conducted in the absence of any commercial or financial relationships that could be construed as a potential conflict of interest.

## Publisher’s note

All claims expressed in this article are solely those of the authors and do not necessarily represent those of their affiliated organizations, or those of the publisher, the editors and the reviewers. Any product that may be evaluated in this article, or claim that may be made by its manufacturer, is not guaranteed or endorsed by the publisher.

## Abbreviations

CHE: Catastrophic health expenditure
CHARLS: China Health and Retirement longitudinal Study
PPS: Probability proportional to size sampling
CTP: Capacity to pay
OOP: Out-of-pocket payment
UEBMI: urban employee basic medical insurance
URRBMI: urban–rural resident medical insurance
PSM: Propensity score matching
ATT: Average treatment effect on the treated
