# Supplementary material for: Catastrophic health expenditure of households with hypertension: a comparative study in China
Source: Front Public Health. 2023 Jun 8;11:1176170. doi: 10.3389/fpubh.2023.1176170 (PMC10285052; doi:10.3389/fpubh.2023.1176170)
Supplement: Supplementary file 1 [file Table_1.docx]

**Supplementary table 1: Differences in covariates before and after matching in subgroups**

| variables | U/M | Only hypertension ***&*** without chronic disease | | | Multimorbidity ***&*** without chronic disease | | |
| --- | --- | --- | --- | --- | --- | --- | --- |
|  |  | STD (%) | t-value | *P* | STD (%) | t-value | *P* |
| Gender | U | 1.1 | 0.27 | 0.786 | -10.2 | -4.29 | <0.001 |
|  | M | -1.9 | -0.38 | 0.701 | -6.7 | -3.18 | 0.001 |
| Age | U | 45.4 | 11.99 | <0.001 | 68.0 | 28.09 | <0.001 |
|  | M | -0.1 | -0.01 | 0.991 | -9.4 | -3.92 | <0.001 |
| Education | U | -17.7 | -4.43 | <0.001 | -25.4 | -10.66 | <0.001 |
|  | M | 1.4 | 0.28 | 0.778 | 6.7 | 3.09 | 0.002 |
| Marital status | U | 15.6 | 4.07 | <0.001 | 27.0 | 11.08 | <0.001 |
|  | M | 2.9 | 0.56 | 0.577 | -3.8 | -1.64 | 0.100 |
| work status | U | -25.5 | -6.62 | <0.001 | -55.9 | -23.04 | <0.001 |
|  | M | -1.0 | -0.20 | 0.844 | 1.3 | 0.57 | 0.572 |
| Household size | U | -4.6 | -1.14 | 0.253 | -8.4 | -3.51 | <0.001 |
|  | M | -1.9 | -0.37 | 0.713 | -1.6 | -0.77 | 0.444 |
| Health insurance | U | 0.3 | 0.09 | 0.931 | -2.4 | -1.01 | 0.314 |
|  | M | -2.7 | -0.56 | 0.574 | -6.9 | -3.35 | 0.001 |
| Residence | U | -2.8 | -0.71 | 0.480 | 4.2 | 1.75 | 0.080 |
|  | M | 1.2 | 0.24 | 0.808 | 1.5 | 0.71 | 0.476 |
| Disabled members | U | 16.5 | 4.25 | <0.001 | 57.7 | 23.74 | <0.001 |
|  | M | 1.5 | 0.29 | 0.772 | -0.4 | -0.17 | 0.862 |
| Inpatient service | U | 13.0 | 3.48 | 0.001 | 60.0 | 23.62 | <0.001 |
|  | M | 2.3 | 0.42 | 0.675 | 5.7 | 2.25 | 0.024 |
| Outpatient service | U | 6.5 | 1.68 | 0.093 | 39.0 | 15.68 | <0.001 |
|  | M | 0.4 | 0.09 | 0.932 | 5.5 | 2.34 | 0.019 |
| Region | U | -0.0 | -0.00 | 1.000 | 14.7 | 6.19 | <0.001 |
|  | M | 0.2 | 0.05 | 0.964 | -1.6 | -0.74 | 0.462 |
| Economic level | U | -15.5 | -3.93 | <0.001 | -16.9 | -7.09 | <0.001 |
|  | M | 0.5 | 0.10 | 0.924 | 6.8 | 3.20 | 0.001 |

Note: U denotes before matching, M denotes after matching. STD denotes standardized difference The tests in the table were based on radius matching and the radius was set to 0.01.
